# Serological response after COVID-19 infection compared to vaccination against COVID-19 in children with autoimmune rheumatic diseases

**DOI:** 10.1186/s12969-024-01003-0

**Published:** 2024-07-25

**Authors:** Tjaša Šinkovec Savšek, Mojca Zajc Avramovič, Tadej Avčin, Miša Korva, Tatjana Avšič-Županc, Nataša Toplak

**Affiliations:** 1 Department of Allergology, Rheumatology and Clinical Immunology, University Children’s Hospital, University Medical Centre Ljubljana, Ljubljana, Slovenia; 2 Faculty of Medicine, University of Ljubljana, Ljubljana, Slovenia; 3 Institute of Microbiology and Immunology, Faculty of Medicine, University of Ljubljana, Ljubljana, Slovenia

**Keywords:** COVID-19, Vaccination, Serological response, Safety, Efficacy, Paediatric autoimmune rheumatic diseases

## Abstract

**Background:**

Paediatric patients with autoimmune rheumatic diseases (pARD) have a dysregulated immune system, so infections present a major threat to them. To prevent severe COVID-19 infections we aimed to vaccinate them as soon as possible. Studies have shown that the BNT162b2 vaccine is safe, effective, and immunogenic, however, in a short observation period, only.

**Methods:**

The main objective was to compare the serological response between three groups of pARD: after SARS-CoV-2 infection, after vaccination against COVID-19 with two doses of the BNT162b2 vaccine, and after experiencing both events. Data on demographics, diagnosis, therapy, and serology (anti-SARS-CoV-2 IgG/IgA) were collected from March 2020 to April 2022. For statistical analysis ANOVA, Mann-Whitney U test, Chi-square test and Fisher’s exact test were applied. To compare adverse events (AE) after vaccination we included a control group of healthy adolescents.

**Results:**

We collected data from 115 pARD; from 92 after infection and 47 after vaccination. Twenty-four were included in both groups. Serological data were available for 47 pARD after infection, 25 after vaccination, and 21 after both events. Serological response was better after vaccination and after both events compared to after infection only. No effect of medication on the antibody levels was noted. The safety profile of the vaccine was good. Systemic AE after the first dose of the vaccine were more common in healthy adolescents compared to pARD. In the observation period of 41.3 weeks, 60% of vaccinated pARD did not experience a symptomatic COVID-19 infection.

**Conclusions:**

IgG and IgA anti-SARS-CoV-2 levels were higher after vaccination and after both events compared to after infection only. Six months after vaccination we observed an increase in antibody levels, suggesting that pARD had been exposed to SARS-CoV-2 but remained asymptomatic.

**Trial registration:**

The study was approved by the Medical Ethics Committee of the Republic of Slovenia (document number: 0120–485/2021/6).

## Background

Children with autoimmune rheumatic diseases (ARD) have a dysregulated immune system impaired by the disease and immunosuppressive treatment [1]. At the beginning of the Coronavirus disease 2019 (COVID-19) pandemic there was a great concern about the possibility of severe acute respiratory syndrome coronavirus 2 (SARS-CoV-2) infection in these children. Throughout the pandemic, it became clear that the clinical presentation of COVID-19 in children is typically milder and the outcome favourable compared to adults [2]. A similar experience was recorded in children, adolescents, and young adults with ARD at the University Children’s Hospital Ljubljana, Slovenia, where 81% of infections in patients with ARD (pARD) were asymptomatic or mild [3]. Studies conducted in other centres also showed comparable results [4–7]. However, in children without any chronic disease, who got COVID-19, cases of severe disease and/or development of a post-infectious multisystem hyperinflammatory syndrome named multisystem inflammatory syndrome in children have been described [8].

To prevent severe infections and complications and given the contagious nature and rapid spread of SARS-CoV-2, it quickly became clear that the best method of protection against COVID-19 is vaccination [9]. The first vaccine approved for adolescents aged 12–18 years was the BNT162b2 Comirnaty (Pfizer-BioNTech), based on the delivery of messenger RNA encoding the SARS-CoV-2 spike glycoprotein [10]. In a study published by Frenck, Jr. et al. in 2021, the BNT162b2 Comirnaty vaccine was proven to be safe, effective, and immunogenic when used in healthy adolescents [11]. An English study from the EULAR COVAX physician-reported registry from 2022 showed that the vaccine was also safe and effective in adolescents with ARD [12], and later, more studies confirmed that finding [13–16]. An Israeli study showed that the efficacy of the BNT162b2 vaccine in pARD was comparable to healthy controls and that immunomodulatory therapy had no effect on it [17].

Later, researchers performed studies taking the immunogenicity of the vaccine into account. Results showed that the serological response after vaccination against COVID-19 in pARD is sufficient, but immunogenicity is influenced by immunomodulatory therapy. A study done by Akgün et al. noted that all pARD had elevated IgG levels after the second dose of the BNT162b2 Comirnaty vaccine, however, those who were receiving conventional synthetic disease-modifying anti-rheumatic drugs (csDMARDs) and biologic DMARDs (bDMARDs) simultaneously, had significantly lower median levels of the anti-receptor-binding domain (anti-RBD) IgG than those receiving only cDMARDs [18]. In an Israeli study Henshin-Bekenstein et al. compared seropositivity rates between pARD and healthy controls. They noted that not all pARD developed antibodies, and the antibody levels were also significantly lower in pARD compared with controls [13]. An important observation was made in a study by Dimpoulou et al., where they reported a significant decrease in antibody levels in adolescents with juvenile idiopathic arthritis (JIA) 6 months after vaccination [19].

While already published studies report on anti-SARS-CoV-2 IgG antibodies, less is known regarding IgA response in pARD after COVID-19 and vaccination against COVID-19. IgA is the most abundant type of antibody in the body and is mainly found on mucosal surfaces as a dimeric secretory IgA. Mucosal IgA plays an important role in preventing the adherence and invasion of pathogens by its neutralising activity, whereas monomeric, serum IgA, is associated with the activation of the phagocytic immune system [20, 21]. Several studies have shown that IgA response plays an important early neutralising role after SARS-CoV-2 infection [22, 23]. In the evaluation of the immune response to influenza, IgA together with IgG was found to be more important in protecting against secondary infection than IgG and IgM immune responses together [24, 25]. Although IgM is considered the first line of humoral response, a peculiarity of SARS-CoV-2 infection is that all three antibodies, IgA, IgG and IgM, can be detected rapidly after antigen encounter. IgG and IgA can often be detected even before IgM, suggesting that the initial IgM response may be weak [26, 27], and thus measuring the IgA and IgG response may be more sensitive. A study by Padoan et al. also showed that specific IgA responses were detectable in 75% of generally healthy patients after COVID-19 and appeared to be stronger and more persistent than IgM responses [28].

To the best of our knowledge, we report the first data on serological response in pARD after COVID-19 infection compared to vaccination against COVID-19.

## Methods

The aim of the study was to compare the serological response in pARD between three groups: after SARS-CoV-2 infection, after vaccination against COVID-19, and after experiencing both events. The safety and efficacy of the vaccine were also studied. It was a single-centre study conducted between March 2020 and April 2022, at the University Children’s Hospital in Ljubljana, Slovenia.

The pARD were followed prospectively after infection and/or vaccination. Data and blood samples were collected at regular visits at the rheumatology outpatient clinic. The study was approved by the Medical Ethics Committee of the Republic of Slovenia (document number: 0120–485/2021/6). Written informed consent was obtained from parents/caregivers and patients, older than 15 years.

### Study population

The study population included patients with childhood-onset ARD (ages 2–23 years) including JIA, idiopathic uveitis, systemic or juvenile localized scleroderma, systemic lupus erythematosus (SLE), systemic vasculitis, juvenile dermatomyositis, and chronic recurrent multifocal osteomyelitis. Other, less common diagnoses included cryopyrin-associated periodic syndrome, rheumatic fever, and undifferentiated connective tissue disease. Diagnoses were established based on the valid criteria for their respective disease in all patients [29–35].

Based on the criteria they met, pARD were divided in two groups:

- Group 1 (pARD who experienced a COVID-19 infection): laboratory-confirmed SARS-CoV-2 infection with a real-time RT-PCR test or a rapid antigen test or positive serology for IgG anti-SARS-CoV-2 antibodies in pARD with a confirmed contact with SARS-CoV-2.
- Group 2 (pARD who were vaccinated against COVID-19): patients who received two doses of the BNT162b2 Comirnaty vaccine in a span of 3–9 weeks.

For the evaluation and analysis of the serology (anti-SARS-CoV-2 IgG and IgA) pARD were further divided into three subgroups:

- Subgroup 1 (serology in pARD who experienced a COVID-19 infection): serology collected from pARD only after infection; if they got vaccinated after the infection, the later recorded serology values were analysed as part of Subgroup 3.
- Subgroup 2 (serology in pARD who were vaccinated against COVID-19): serology collected from pARD only after vaccination; if they got infected after the vaccination, the later recorded serology values were analysed as part of Subgroup 3.
- Subgroup 3 (serology in pARD who experienced both events – COVID-19 infection and vaccination against COVID-19): serology collected from pARD after they experienced both events.

For the evaluation of the safety profile of BNT162b2 Comirnaty vaccine we included a control group consisting of healthy adolescents. To be included they had to meet the inclusion criteria:

- Between the ages of 14 and 19 years.
- No ARD diagnosis.
- At least two doses of the BNT162b2 Comirnaty vaccine received in a time span of 3–9 weeks.

### Serology for IgG and IgA anti-SARS-CoV-2 antibodies

For the confirmation of acute SARS-CoV-2 infection, real-time reverse transcription-polymerase chain reaction (RT-PCR) assays were used. In cases where individuals had a confirmed contact with SARS-CoV-2 and appeared asymptomatic, serology confirmed the diagnosis of infection [36]. Serologic testing, measuring of the anti-SARS-CoV-2 IgG and IgA antibodies was performed in Groups 1 and 2 at different time points, namely: up to 3 months, 3–6 months, 6–12 months, and more than 12 months after the event. The serologic measurement was conducted with the Anti-SARS-CoV-2 ELISA (IgG and IgA) EUROIMMUN kit from Lübeck, Germany, following the manufacturer’s instructions and as previously described [37]. The test uses an indirect enzyme-linked immunosorbent assay, which involves the specific interaction between viral antigens bound to a solid support (polystyrene microtiter plate with wells) and specific antiviral antibodies present in the subject’s serum. The antigen used in the Anti-SARS-CoV-2 ELISA IgG and IgA test is the recombinant protein of the S1 subunit, specifically the S spike protein of the SARS-CoV-2 virus. This protein is known to be the most immunodominant and specific part of SARS-CoV-2 [37]. A sample is considered positive for the presence of anti-SARS-CoV-2 IgG or IgA antibodies when the calculated value is equal to or greater than 1.1, and negative when the value is less than 0.8. Samples with intermediate values ​​(between 0.8 and 1.1) are defined as “threshold values” [37, 38].

### Safety of the BNT162b2 comirnaty vaccine

The safety of the BNT162b2 Comirnaty vaccine was assessed in both study (pARD) and control group (healthy adolescents) using a specific questionnaire after each dose of the vaccine. Participants were asked about local (pain, redness, swelling, itching, and tingling at the vaccination site) and systemic (nausea, vomiting, runny nose, cough, myalgia, arthralgia, fever above 38 °C, chills, feeling unwell, headache, tiredness, and weakness) adverse events (AE). They were also inquired about the possible allergic reaction and/or hospitalization after each dose of the vaccine.

### Efficacy of the BNT162b2 comirnaty vaccine in pARD

To evaluate the efficacy of the BNT162b2 Comirnaty vaccine in pARD, patients were asked to inform their attending paediatric rheumatologist through e-mail or phone call in case they tested positive or had a confirmed contact and displayed symptoms of COVID-19. Additionally, possible SARS-CoV-2 infection was actively discussed at every regular visit at the rheumatology outpatient clinic until April 2022.

### Statistical analysis

Statistical analysis was performed using the IBM SPSS program (version 29.0.2.0). To test for the differences between continuous variables the ANOVA, and for non-parametric data Mann-Whitney U test was used. For categorical variables, the Chi-square test or Fisher’s exact test was used, and a *p*-value ≤ 0.05 was considered statistically significant.

## Results

### Study population

We gathered data from 115 pARD and divided them into two groups. Group 1 consisted of 92 pARD after SARS-CoV-2 infection, and Group 2 of 47 pARD after vac-cination against COVID-19. Since 24 patients had COVID-19 before or after receiving the vaccine, we included them in both groups. Therefore, in Group 1, we included pARD who only had COVID-19 (*n* = 68) and pARD after both events (*n* = 24), and in Group 2, we included pARD only after vaccination (*n* = 23) and pARD after both events (*n* = 24). The division into Groups 1 and 2 was used for the analysis of disease relapse rate in pARD. Results are already published and available in an open-access format online [3]. We determined the effectiveness of the vaccine and adverse events (AE) within Group 2.

**Fig. 1:**
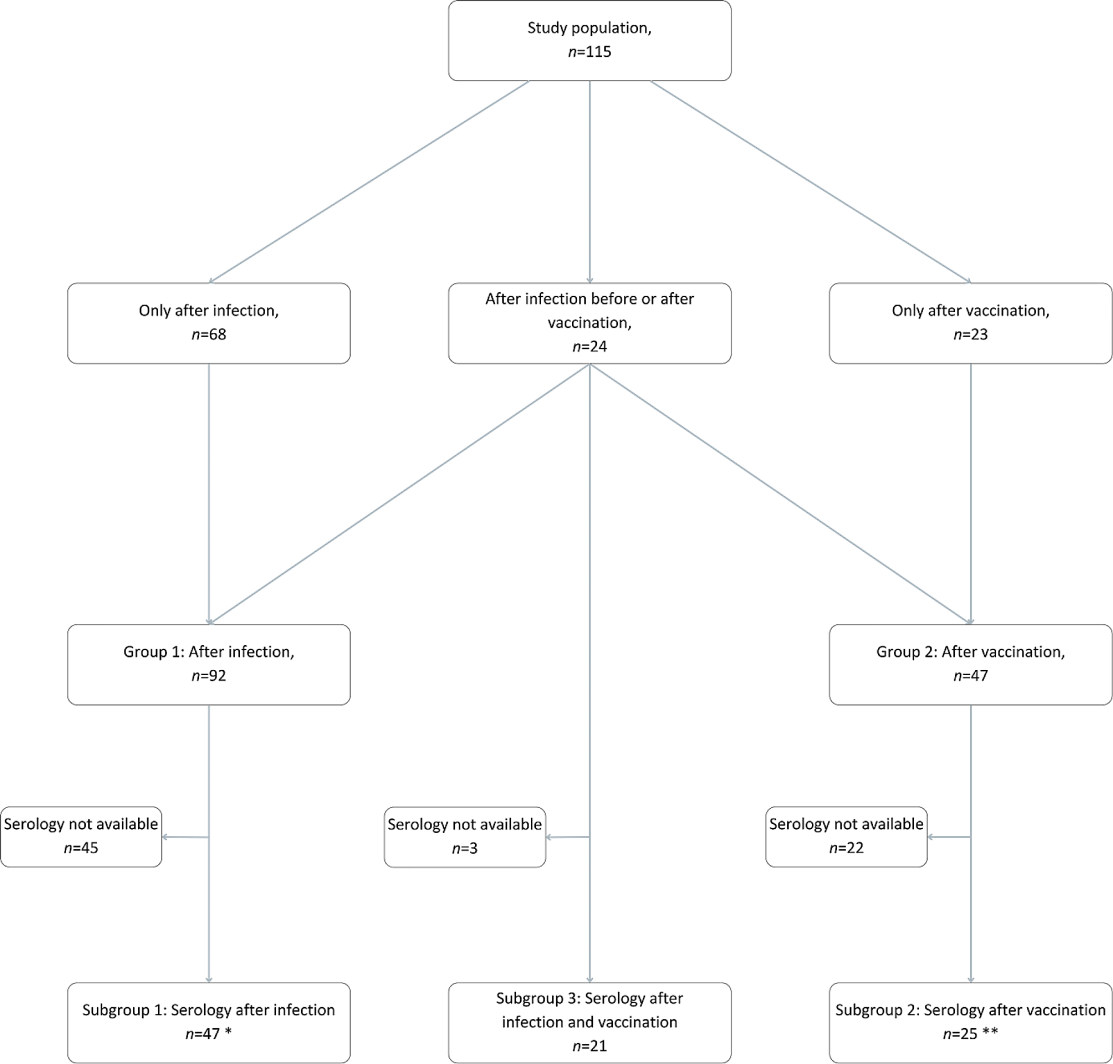
Graphical representation of the study population * Subgroup 1: of the pARD who got vaccinated after the infection, only IgG/IgA values after infection but before vaccination are included – as soon the infected pARD (Subgroup 1) got vaccinated, they were considered after both events (Subgroup 3) and their later recorded IgG/IgA values were analysed as part of Subgroup 3 ** Subgroup 2: of the pARD who got infected after the vaccination, only IgG/IgA values after vaccination but before infection are included – as soon as the vaccinated pARD (Subgroup 2) got infected, they were considered after both events (Subgroup 3) and their later recorded IgG/IgA values were analysed as part of Subgroup 3

To understand the serological response after infection or vaccination, pARD were further divided into three subgroups based on the event (infection, vaccination, or both) they experienced. Serological data were available for 47 of 92 (51%) pARD after infection (Subgroup 1) and for 25 of 47 (53%) after vaccination (Subgroup 2). For the analysis of Subgroups 1 and 2, only values after one event (infection or vaccination) were included. The serological data obtained from patients who were vaccinated before or after the in-fection (experienced both events, *n* = 24) were considered part of Subgroup 3 after they experienced the second event. In Subgroup 3, serological data was available for 21 of 24 pARD (88%). A graphical representation of the study population with divisions into groups and subgroups is available in Fig. 1.

The basic characteristics of the groups and subgroups are presented in the text below.

The majority of pARD had JIA (81% in Group 1, 86% in Group 2). The mean age was 13.4 (S.D.=4.1) years in Group 1, and 15.9 (S.D.=2.4) years in Group 2; there were 73% females in Group 1, and 64% in Group 2.

In Group 1, 69 (75%) and in Group 2, 32 (68%) of pARD were receiving immunomodulatory medications. Of those in Group 1, 23 (33%) pARD were using csDMARDs, 30 (43%) were using bDMARDs, and 16 (23%) were receiving a combination of bDMARDs and csDMARDs. Of those in Group 2, 10 (31%) were using csDMARDs, 10 (31%) were using bDMARDs, and 12 (38%) were receiving a combination of bDMARDs and csDMARDs.

More detailed information regarding groups 1 and 2 (diagnoses, therapy, etc.) has already been published and is available in an open-access format online in Tables 1 and 2 [3].

To evaluate the safety profile of the BNT162b2 Comirnaty vaccine in pARD, we also collected data from 92 healthy adolescents (63% females) who were vaccinated with two doses of the BNT162b2 Comirnaty vaccine. The mean age was 16.3 (S.D.=1.3) years at the time of vaccination. Four (4%) adolescents reported having a chronic condition; one had asthma, and three had type 1 diabetes. All 92 had no symptoms of acute disease before receiving the vaccine.

In 47 pARD, a total of 94 vaccinations were performed. On average, patients received the second dose 3.7 (S.D.=1.4) weeks after the first dose. In 92 healthy adolescents, a total of 184 vaccinations were performed. Data on the time interval between the first and second dose was unfortunately not available.

In 92 pARD, we registered 103 COVID-19 cases, 11 patients got the infection twice. Infection was confirmed by a positive RT-PCR or rapid antigen test for SARS-CoV-2 in 65 (63%) cases. In 27 (26%) cases, pARD were in contact with a SARS-CoV-2-positive person and had a typical clinical presentation of COVID-19. In the other 11 (11%) cases, COVID-19 was confirmed retrospectively by positive IgG anti-SARS-CoV-2 antibodies. A more in-depth description regarding COVID-19 confirmation has already been published and is available in an open-access format online [3].

### Serological response after COVID-19 infection and vaccination

Basic characteristics for the three subgroups of pARD are available in Table 1.

**Table 1:** Basic characteristics of patients with available serological data

Characteristics	Subgroup 1^*a*^ (*n* = 47)	Subgroup 2^*b*^ (*n* = 25)	Subgroup 3^c^ (*n* = 21)
Mean age (S.D.) in years	12.1 (4.3)	15.5 (2.0)	15.8 (2.7)
≥ 12 years, *n* (%)	25 (53)	25 (100)	21 (100)
Females, *n* (%)	37 (79)	15 (60)	15 (71)
**Diagnosis**			
JIA^*d*^, *n* (%)	41 (88)	22 (88)	18 (85)
SLE^*e*^ and vasculitis, *n* (%)	2 (4)	0 (0)	0 (0)
Uveitis, *n* (%)	1 (2)	0 (0)	0 (0)
CRMO^*f*^, *n* (%)	0 (0)	2 (8)	1 (5)
jL^*g*^, *n* (%)	2 (4)	1 (4)	1 (5)
Other^*h*^, *n* (%)	1 (2)	0 (0)	1 (5)

^*a*^ Subgroup 1 – pARD after infection with available serological data, ^*b*^ Subgroup 2 – pARD after vaccination with available serological data, ^*c*^ Subgroup 3 – pARD after infection and vaccination with available serological data, ^*d*^ JIA – juvenile idiopathic arthritis, ^*e*^ SLE – systemic lupus erythematosus, ^*f*^ CRMO – chronic recurrent multifocal osteomyelitis, ^*g*^ jLS – juvenile localised scleroderma, ^*h*^Other – undifferentiated connective tissue disease

In Subgroup 1, 35 (74%) pARD were on immunomodulatory medications (Table 2). Of those, 28 (80%) were receiving one, 6 (17%) two, and 1 (3%) three medications. Twelve (26%) pARD were not taking any medications at the time of SARS-CoV-2 infection.

In Subgroup 2, 15 (60%) pARD were on immunomodulatory medications (Table 2). Of those, 10 (67%) were receiving one, and five (33%) two medications. Ten (40%) pARD were not taking any medications at the time of vaccination against COVID-19.

In Subgroup 3, 15 (71%) pARD were on immunomodulatory medications (Table 2). Of those, 9 (60%) were receiving one, and six (40%) two medications. Six (29%) were not taking any medications at the time of the second event (infection or vaccination).

**Table 2:** Medications among patients with available serological data

Medication	Medications in Subgroup 1^*a*^ (*n* = 47)	Medications in Subgroup 2^*b*^ (*n* = 25)	Medications in Subgroup 3^*c*^ (*n* = 21)
No medications	12 (26)	10 (40)	6 (29)
GCS^*d*^, *n* (%)	3 (6)	0 (0)	0 (0)
**csDMARDs** ^*e*^			
Hydroxychloroquine, *n* (%)	2 (4)	0 (0)	0 (0)
Methotrexat, *n* (%)	10 (21)	7 (28)	4 (19)
Leflunomide, *n* (%)	0 (0)	2 (8)	2 (10)
Mycophenolate mofetil, *n* (%)	1 (2)	1 (4)	1 (5)
Cyclosporine, *n* (%)	1 (2)	0 (0)	0 (0)
Azathioprine, *n* (%)	1 (2)	0 (0)	0 (0)
**bDMARDs** ^*f*^			
TNFα inhibitors, *n* (%)	19 (40)	9 (36)	10 (48)
IL-6 inhibitors, *n* (%)	0 (0)	1 (4)	1 (5)
IL-1 inhibitors, *n* (%)	2 (4)	0 (0)	1 (5)
Rituximab, *n* (%)	1 (2)	0 (0)	0 (0)
Abatacept, *n* (%)	2 (4)	0 (0)	0 (0)
**tsDMARDs** ^*g*^			
Baricitinib, *n* (%)	1 (2)	0 (0)	1 (5)

^*a*^ Subgroup 1 – pARD after infection with available serological data, ^*b*^ Subgroup 2 – pARD after vaccination with available serological data, ^*c*^ Subgroup 3 – pARD after infection and vaccination with available serological data, ^*d*^ GCS – glucocorticosteroids, ^*e*^ csDMARDs – conventional synthetic disease-modifying anti-rheumatic drugs, ^*f*^ bDMARDs – biologic disease-modifying anti-rheumatic drugs, ^*g*^ tsDMARDs – targeted synthetic disease-modifying anti-rheumatic drugs

We confirmed the anti-SARS-CoV-2 antibodies in 40 (85%) pARD from Subgroup 1, in 25 (100%) from Subgroup 2, and in 21 (100%) from Subgroup 3. To compare the mean levels of IgA and IgG antibodies between the three groups in three different time frames (less than three months, three to six months, and six to 12 months), we performed one-way ANOVA.

#### IgG antibody levels

There was a statistically significant difference in IgG levels between the three Subgroups as determined by one-way ANOVA for all time frames (less than three months, three to six months, and six to 12 months). The results were as follows:

For less than three months after infection, vaccination, or the second event (in Subgroup 3): F(2,53) = 55.70, *p* < 0.001. A Tukey post hoc test revealed that IgG antibody levels were statistically significantly lower in Subgroup 1 (2.61 ± 2.83) compared to Subgroup 2 (8.84 ± 2.80, *p* < 0.001) and Subgroup 3 (10.12 ± 1.92, *p* < 0.001). There was no statistically significant difference in IgG levels between Subgroups 2 and 3 (*p* = 0.43).

For three to six months after infection, vaccination, or the second event (in Subgroup 3): F(2,29) = 16.43, *p* < 0.001. A Tukey post hoc test revealed that IgG antibody levels were statistically significantly lower in Subgroup 1 (2.82 ± 2.23) compared to Subgroup 2 (6.99 ± 2.90, *p* < 0.001) and Subgroup 3 (9.59 ± 0.57, *p* < 0.001). Again, there was no statistically significant difference in IgG levels between Subgroups 2 and 3 (*p* = 0.24).

For six to 12 months after infection, vaccination, or the second event (in Subgroup 3): F(2,29) = 19.68, *p* < 0.001. A Tukey post hoc test revealed that IgG antibody levels were statistically significantly lower in Subgroup 1 (2.61 ± 2.63) compared to Subgroup 2 (8.61 ± 3.33, *p* < 0.001) and Subgroup 3 (9.93 ± 1.24, *p* < 0.001). Again, there was no statistically significant difference in IgG levels between Subgroups 2 and 3 (*p* = 0.76).

#### IgA antibody levels

Similar results were obtained for IgA antibodies with a statistically significant difference in IgA levels between the three Subgroups as determined by one-way ANOVA for all time frames (less than three months, three to six months, and six to 12 months).

For less than three months after infection, vaccination, or the second event (in Subgroup 3): F(2,53) = 32.39, *p* < 0.001. A Tukey post hoc test revealed that IgA antibody levels were statistically significantly lower in Subgroup 1 (1.49 ± 2.12) compared to Subgroup 2 (5.84 ± 4.72, *p* = 0.008) and Subgroup 3 (10.08 ± 4.60, *p* < 0.001). There was a statistically significant difference in IgA levels between Subgroups 2 and 3 (*p* = 0.014).

For three to six months after infection, vaccination, or the second event (in Subgroup 3): F(2,29) = 4.02, *p* = 0.029. A Tukey post hoc test revealed that IgA antibody levels were statistically significantly lower in Subgroup 1 (2.29 ± 2.67) compared to Subgroup 3 (7.00 ± 4.78, *p* = 0.023), but no statistically significant difference was noted when compared to Subgroup 2 (2.68 ± 2.00, *p* = 0.92). There was a statistically significant difference in IgA levels between Subgroups 2 and 3 (*p* = 0.049).

For six to 12 months after infection, vaccination, or the second event (in Subgroup 3): F(2,29) = 4.43, *p* = 0.021. A Tukey post hoc test revealed that IgA antibody levels were statistically significantly lower in Subgroup 1 (2.41 ± 2.90) compared to Subgroup 2 (7.49 ± 6.33, *p* = 0.018), but no statistically significant difference was noted when compared to Subgroup 3 (5.97 ± 5.20, *p* = 0.43). Again, we found no statistically significant difference in IgA levels between Subgroups 2 and 3 (*p* = 0.87).

**Fig. 2:**
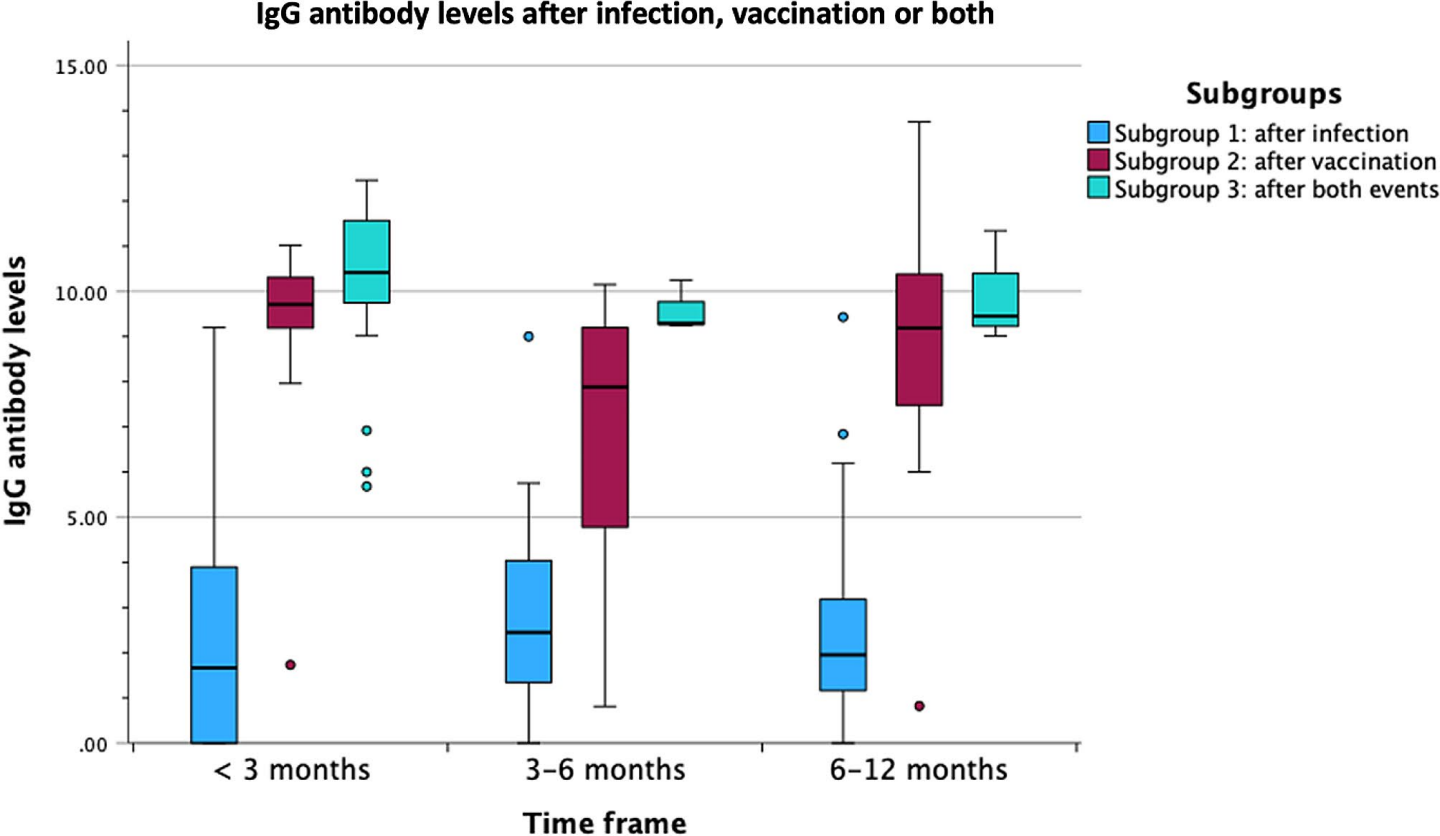
IgG antibody levels after COVID-19 infection, vaccination or both events

**Fig. 3:**
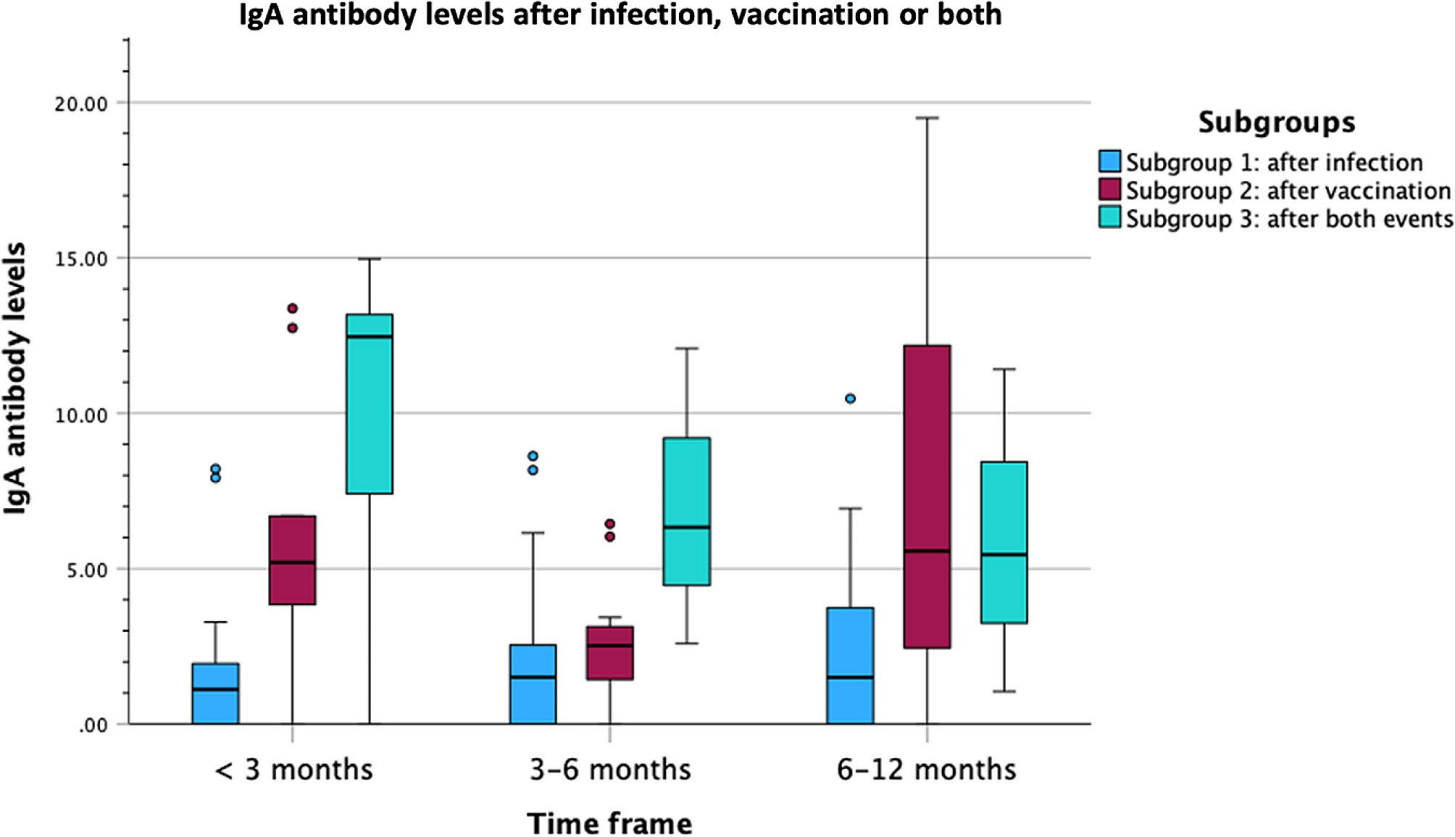
IgA antibody levels after infection, vaccination or both COVID-19 infection, vaccination or both events

A graphical representation of the results is available in Figs. 2 and 3.

### Effect of medications on IgG and IgA response

A series of Mann-Whitney U tests were conducted to determine whether there is a difference in IgG and IgA levels between pARD who were receiving methotrexate (MTX) or TNF-α inhibitors at the time of infection, vaccination, or the second event (Subgroup 3) and those who were not. Tests were carried out for Subgroups 1 and 2 for the three time frames (less than three months, three to six months, and six to 12 months), and for Subgroup 3 for one time frame only (less than three months), since not enough data were available to also do the test for the other two time frames (three to six months and six to 12 months). In total, for each medication 14 tests were performed, therefore, the Benjamini and Hochberg method to control the false discovery rate when doing multiple comparisons was used, and for this part of the analysis a *p*-value < 0.0036 was considered statistically significant.

The results indicated a non-significant difference between pARD who were taking MTX compared to those who were not for all Subgroups and observed time frames (*p* > 0.0036). Similarly, no statistically significant difference was noted between pARD who were receiving TNF-α inhibitors compared to those who were not for all Subgroups and observed time frames (*p* > 0.0036).

### Safety of the BNT162b2 comirnaty vaccine

We received completed questionnaires regarding the safety of the vaccine from 43 pARD (91%) and 92 healthy adolescents (100%) after vaccination with the BNT162b2 Comirnaty vaccine. The basic characteristics of healthy adolescents are reported in Table 3.

**Table 3:** Basic characteristics of healthy adolescents

Characteristics	Healthy adolescents (*n* = 92)
Mean age (S.D.) in years	16.3 (1.3)
≥ 12 years, *n* (%)	92 (100)
Females, *n* (%)	58 (63)
**Diagnosis of a chronic disease**	
Yes, *n* (%)	4 (4)^a^

^*a*^ Three adolescents reported having type 1 diabetes, one adolescent reported having asthma

Results of reported AE after the first and the second dose of the BNT162b2 Comirnaty vaccine for pARD and healthy adolescents are available in Table 4.

**Table 4:** Adverse events after the first dose of the BNT162b2 Comirnaty vaccine in healthy adolescents and patients with autoimmune rheumatic diseases

	After the first dose of the vaccine, *n* (%)					After the second dose of the vaccine, *n* (%)				
	pARD^a^		Healthy adolescents			pARD		Healthy adolescents		
AE^*b*^	Yes	No	Yes	No	*p*-value	Yes	No	Yes	No	*p*-value
AE – overall	38 (88)	5 (12)	87 (95)	5 (5)	0.20	40 (93)	3 (7)	83 (90)	9 (10)	0.59
AE – local	37 (86)	6 (14)	83 (90)	9 (10)	0.47	36 (84)	7 (16)	73 (79)	19 (21)	0.55
AE – systemic	20 (47)	23 (53)	60 (65)	32 (35)	**0.039**	29 (67)	14 (33)	63 (68)	29 (32)	0.9
**Local AE**										
Pain at VS^*c*^	37 (86)	6 (14)	80 (87)	12 (13)	0.88	36 (84)	7 (16)	71 (77)	21 (23)	0.38
Redness at VS	3 (7)	40 (93)	8 (9)	84 (91)	0.73	3 (7)	40 (93)	7 (8)	85 (92)	0.9
Swelling at VS	10 (23)	33 (77)	13 (14)	79 (86)	0.19	8 (19)	35 (81)	14 (15)	78 (85)	0.62
Itching at VS	5 (12)	38 (88)	12 (13)	80 (87)	0.82	2 (5)	41 (95)	5 (5)	87 (95)	0.85
Tingling at VS	0 (0)	43 (100)	5 (5)	87 (95)	0.18*	0 (0)	43 (100)	5 (5)	87 (95)	0.18*
**Systemic AE**										
Nausea	3 (7)	40 (93)	13 (14)	79 (86)	0.23	4 (9)	39 (91)	13 (14)	79 (86)	0.43
Vomiting	0 (0)	43 (100)	0 (0)	92 (100)	1*	0 (0)	43 (100)	1 (1)	91 (99)	1*
Runny nose	0 (0)	43 (100)	5 (5)	87 (95)	0.18*	3 (7)	40 (93)	5 (5)	87 (95)	0.72
Cough	0 (0)	43 (100)	3 (3)	89 (97)	0.55*	0 (0)	43 (100)	3 (3)	89 (97)	0.55*
Myalgia	8 (19)	35 (81)	29 (32)	63 (68)	0.12	13 (30)	30 (70)	28 (30)	64 (70)	0.98
Arthralgia	2 (5)	41 (95)	6 (7)	86 (93)	0.67	6 (14)	37 (86)	9 (10)	83 (90)	0.47
Fever > 38 °C	1 (2)	42 (98)	6 (7)	86 (93)	0.31	6 (14)	37 (86)	12 (13)	80 (87)	0.88
Chills	2 (5)	41 (95)	10 (11)	82 (89)	0.24	7 (16)	36 (84)	15 (16)	77 (84)	1
Feeling unwell	8 (19)	35 (81)	30 (33)	62 (67)	0.092	19 (44)	24 (56)	36 (39)	56 (61)	0.58
Headache	8 (19)	35 (81)	18 (20)	74 (80)	0.9	18 (42)	25 (58)	31 (34)	61 (66)	0.36
Tiredness	13 (30)	30 (70)	37 (40)	55 (60)	0.26	18 (42)	25 (58)	58 (63)	34 (37)	**0.021**
Weakness	5 (12)	38 (88)	32 (35)	60 (65)	**0.0050**	11 (26)	32 (74)	40 (43)	52 (57)	**0.046**
Allergic reaction	0 (0)	43 (100)	0 (0)	92 (100)	1*	0 (0)	43 (100)	0 (0)	92 (100)	1*
Hospitalization	0 (0)	43 (100)	0 (0)	92 (100)	1*	0 (0)	43 (100)	0 (0)	92 (100)	1*

^*a*^ pARD – patients with autoimmune rheumatic diseases, ^*b*^ AE – adverse events, ^*c*^ VS – vaccination site, * for analysis Fisher’s exact test was used

To evaluate the overall frequency of AE between pARD from Group 2 and healthy adolescents, a Chi-square test was performed. The results showed no statistically significant difference between the two groups, with *p*-values of 0.20 after the first dose and 0.59 after the second dose of the vaccine. Furthermore, we compared the frequency of local and systemic AE between the two groups. There were no statistically significant differences in local AE, with *p*-values of 0.47 after the first and 0.55 after the second dose of the vaccine. However, systemic AE were more frequently observed in healthy adolescents after the first (*p* = 0.039) but not after the second dose of the vaccine (*p* = 0.90).

Next, a series of Chi-square tests and Fisher’s exact tests (when at least one of the observed values was zero) were executed to evaluate the frequency of each adverse event between the two groups. *P*-values of < 0.05 were calculated for tiredness after the second dose (*p* = 0.021) and for weakness after both doses of the vaccine (*p* = 0.0050 after the first and 0.046 after the second dose of the vaccine, respectively). However, after using the Benjamini and Hochberg method to control the false discovery rate when applying multiple comparisons, none of the results were statistically significant anymore.

### Efficacy of the BNT162b2 comirnaty vaccine

After getting vaccinated against COVID-19, pARD were followed, on average, for 38.3 (S.D.=11.1) weeks after receiving the second dose. The median follow-up time was 41.3 (IQR 31.3–45.8) weeks. Nineteen (40%) pARD reported having COVID-19 even though they were fully vaccinated, on average 19.2 (S.D.=7.4) weeks after receiving the second dose of the BNT162b2 Comirnaty vaccine. Of those, 16 (84%) had a mild, and three (16%) had a moderate clinical presentation of the disease. None required hospitalization because of COVID-19. In pARD who were vaccinated and still got COVID-19, we registered three (16%) patients who experienced a relapse of their ARD after infection. More detailed information about the clinical presentation of COVID-19, and the relapse rate of the ARD after COVID-19 and vaccination against COVID-19 has already been published. Data is available in an open-access format online [3].

## Discussion

This prospective single-centre study focused on the serological response after COVID-19 and vaccination against COVID-19 and the safety and long-term efficacy of the BNT162b2 Comirnaty vaccine in pARD. We included children, adolescents, and young adults with ARD. At the time of publication of this manuscript, no studies are available comparing serological responses in pARD after COVID-19 and after vaccination against COVID-19. A few studies have been published reporting on the safety and efficacy of the BNT162b2 Comirnaty vaccine, but in a shorter observation period [12, 13, 15–18]. Therefore, this study represents a valuable contribution to the field of paediatric rheumatology.

### Serological response after COVID-19 infection and vaccination

We confirmed IgG and IgA antibodies in 85% of pARD after infection (Subgroup 1), 100% of pARD after vaccination against COVID-19 (Subgroup 2), and 100% after both events (Subgroup 3). We observed an expected drop in IgG and IgA levels three months after pARD from Subgroup 2 received the second dose of vaccine. Interestingly, six months after the second dose, levels of antibodies increased again. We recorded COVID-19 infections in some pARD from Subgroup 2, and they all presented with mild or moderate clinical presentation. As described, serological data of patients from Subgroup 2 after the second event – infection, were analysed as a part of Subgroup 3 (after both events). The pARD from Subgroup 2 have likely been in contact with SARS-COV-2 but did not develop symptoms of the disease, because they were protected by the vaccine. Nevertheless, the contact served as a booster, and the antibody levels increased. In subgroup 3 (after both events), we noted an expected drop in IgG and IgA levels throughout the observed period.

In a multicentre study conducted by Henshin-Bekenstein et al., the seropositivity rate was 97.3% in pARD and 100% in healthy adolescents after vaccination with two doses of the BNT162b vaccine [13]. They noted that the anti-S1/S2 antibody levels were significantly lower in pARD compared to controls (*p* < 0.001). There was a trend towards reduced humoral response in patients treated with mycophenolate mofetil (MMF).

In a study by Yeo et al., humoral immunogenicity was assessed at 2–3 and 4–6 weeks after the first and the second vaccination [14]. They recorded 65% and 99% seropositivity in pARD after the first and second dose, respectively. An increased risk for seronegativity was observed if pARD were treated with MMF or MTX. Similar results were found in a study by Akgün et al. [18].

In a study by Udaondo et al., they compared humoral and cellular immunity after vaccination with the BNT162b2 vaccine between pARD on immunosuppressive treatment (*n* = 40) and a healthy control group (*n* = 24) [39], and observed no difference. Interestingly, they observed a better T-cell response in pARD treated with TNF-α inhibitors compared to the rest of the group, with *p* = 0.012.

In our study no statistically significant effect of MTX or TNF-α inhibitors on IgG or IgA antibody response was observed, so our findings differ from some of the published data. Unfortunately, we only had two pARD on MMF, one in Subgroup 1 and one in Subgroup 2; and the serological data from the patient from Subgroup 2 was analysed as part of Subgroup 3 after he experienced the second event (infection). Therefore, we could not draw any conclusions about the effect of MMF on the antibody levels in our patients. The one pARD on MMF after infection was seropositive with high IgG and IgA antibody levels (10.25 and 2.59, respectively). The patient from Subgroup 2 had positive IgG antibodies (1.73) and negative IgA antibodies after the vaccination, and after the second event (infection), his IgG antibodies increased (6.92), however, his IgA response remained undetected.

### Safety of the BNT162b2 comirnaty vaccine

In our study, there was no statistically significant difference in the overall frequency of AE and the frequency of local AE between pARD and healthy adolescents after vaccination against COVID-19. We did note a statistically significant difference in the frequency of systemic AE between the two groups, with systemic AE more frequently observed in healthy adolescents after the first (*p* = 0.039) but not after the second dose of the vaccine (*p* = 0.90). These results are comparable to already published results [12–14, 16].

AE were mostly mild and transient. Some studies reported a few patients with serious AE [12, 13]; however, our study showed no such cases. As previously described, the BNT162b2 Comirnaty vaccine is safe to use in pARD.

### Efficacy of the BNT162b2 comirnaty vaccine

The main advantage of our study was a relatively long observation period, with pARD being followed, on average, for 38.3 (S.D.=11.3) weeks after receiving the second dose of vaccine. During this time, 19 (40%) pARD got infected with SARS-CoV-2, on average 19.2 (S.D.=7.4) weeks after the second dose. All had a mild or moderate clinical presentation, as defined in an already-published article [3].

Henshin-Bekenstein et al. observed no COVID-19 cases among pARD or the control group during the three months post-vaccine follow-up [13]. Yeo et al. also noted no symptomatic COVID-19 cases in pARD six weeks after vaccination [14] and Lawson-Tovey et al. had a similar experience with no SARS-CoV-2 infections post-vaccination with the median observation period of 7.2 weeks after the first, and 6.3 weeks after the second dose of the vaccine [12]. In a study done by Dimopoulou et al., they confirmed some COVID-19 cases; 5 (24%) pARD got infected after receiving both doses of the BNT162b2 vaccine, on average 19.4 (S.D.=4.6) weeks after the second dose [19]. This information is consistent with our results.

In a study done by Ziv et al., the effectiveness of the vaccine was similar in a large group of adolescents with ARD and healthy controls after the second and third dose of the vaccine [17]. In the median observation period of 19 weeks, they observed 2.7% of COVID-19 cases among pARD and 2.6% among healthy controls after two doses of the vaccine. Therapy had no effect on vaccine effectiveness.

Compared to Ziv et al. we observed a much higher percentage of COVID-19 cases in pARD after being vaccinated with two doses of the BNT162b2 vaccine, 40% vs. 2.7%, however in a much longer observation time, which was 41.3 weeks in our study.

Our study has some limitations. The study sample is smaller than in some other studies, and there is an age difference in the pARD enrolled in the study, with pARD in Group 1 (after infection) aged from 2 to 23 years and pARD in Group 2 (after vaccination) aged from 10 to 21 years. There is also a difference in the age interval in the control group, with healthy adolescents aged from 14 to 19 years. In our study, we relied on the patients to report a COVID-19 infection and obtained an epidemiological history considering a possible COVID-19 infection among the family members. This is especially important in Group 2 (after vaccination); since we did not test for antibodies against the nucleocapsid virus protein N, we cannot be certain, whether the patients were exposed to SARS-CoV-2 before vaccination.

The strength of our study is an extended observation period compared to the other published studies. Additionally, to our knowledge, this is the first study to compare the serological response (IgG and IgA) in paediatric patients after COVID-19 and after vaccination against COVID-19. Furthermore, we were able to contribute additional data on the long-term effectiveness of the BNT162b2 vaccine.

## Conclusions

The serological response was better after vaccination against COVID-19 and after experiencing both events (vaccination and infection) compared to SARS-CoV-2 infection in pARD. We found no effect of MTX or TNF-α inhibitors on IgG and IgA levels in our study group. We observed a good safety profile of the BNT162b2 vaccine in pARD. Systemic AE were more frequent in healthy adolescents after the first dose of the vaccine. The vaccine proved to be effective. In the observation period of 41.3 (IQR 31.3–45.8) weeks, 60% of pARD who received both doses of the BNT162b2 vaccine did not experience a symptomatic SARS-CoV-2 infection. However, the observed trends in IgG and IgA response in pARD after vaccination, with antibodies first decreasing over time as expected, and then increasing again six months after the vaccination, suggest that patients had been exposed to SARS-CoV-2 but did not experience a symptomatic infection. This furthermore supports the positive effect of the vaccination in pARD.

## Data Availability

The datasets used and/or analysed during the current study are available from the corresponding author on reasonable request.

## Abbreviations

AE: Adverse events
Anti-RBD: Anti-receptor-binding domain
ARD: Autoimmune rheumatic diseases
bDMARDs: Biologic disease-modifying anti-rheumatic drugs
COVID-19: Coronavirus disease 2019
csDMARDs: Conventional synthetic disease-modifying anti-rheumatic drugs
JIA: Juvenile idiopathic arthritis
pARD: Paediatric patients with autoimmune rheumatic diseases
SARS-CoV-2: Severe acute respiratory syndrome coronavirus 2
SLE: Systemic lupus erythematosus
TNF-α: Tumour necrosis factor alpha

## References

[1] Makowska J, Styrzyński F. Between COVID-19 severity and its prevention - what should rheumatologists be aware of? Reumatologia. 2021;59(1):1–2. 33707788 10.5114/reum.2021.103941 PMC7944955

[2] Patel NA, Pediatric. COVID-19: systematic review of the literature. Am J Otolaryngol. 2020;41(5):102573. 32531620 10.1016/j.amjoto.2020.102573 PMC7833675

[3] Šinkovec Savšek T, Zajc Avramovič M, Avčin T, Korva M, Avšič Županc T, Toplak N. Disease relapse rate in children with autoimmune rheumatic diseases after COVID-19 infection and vaccination. Pediatr Rheumatol Online J. 2023;21(1):46. 37208721 10.1186/s12969-023-00829-4 PMC10197037

[4] Wakiguchi H, Kaneko U, Sato S, Imagawa T, Narazaki H, Miyamae T. Clinical features of COVID-19 in pediatric rheumatic diseases: 2020–2022 survey of the Pediatric Rheumatology Association of Japan. Viruses. 2023;15(5):1205. 37243292 10.3390/v15051205 PMC10221643

[5] Villacis-Nunez DS, Rostad CA, Rouster-Stevens K, Khosroshahi A, Chandrakasan S, Prahalad S. Outcomes of COVID-19 in a cohort of pediatric patients with rheumatic diseases. Pediatr Rheumatol. 2021;19(1):94. 10.1186/s12969-021-00568-4 PMC8215630 34154620

[6] Kearsley-Fleet L, Chang ML, Lawson-Tovey S, Costello R, Fingerhutová Š, Švestková N, et al. Outcomes of SARS-CoV-2 infection among children and young people with pre-existing rheumatic and musculoskeletal diseases. Ann Rheum Dis. 2022;81(7):998–1005. 35338032 10.1136/annrheumdis-2022-222241 PMC8977459

[7] Sozeri B, Ulu K, Kaya-Akça U, Haslak F, Pac-Kisaarslan A, Otar-Yener G, et al. The clinical course of SARS-CoV-2 infection among children with rheumatic disease under biologic therapy: a retrospective and multicenter study. Rheumatol Int. 2022;42(3):469–75. 34570263 10.1007/s00296-021-05008-w PMC8475421

[8] Feldstein LR, Rose EB, Horwitz SM, Collins JP, Newhams MM, Son MBF, et al. Multisystem inflammatory syndrome in U.S. children and adolescents. N Engl J Med. 2020;383(4):334–46. 32598831 10.1056/NEJMoa2021680 PMC7346765

[9] Verdecia M, Kokai-Kun JF, Kibbey M, Acharya S, Venema J, Atouf F. COVID-19 vaccine platforms: delivering on a promise? Hum Vaccin Immunother. 2021;17(9):2873–93. 34033528 10.1080/21645515.2021.1911204 PMC8381795

[10] Park JW, Lagniton PNP, Liu Y, Xu RH. mRNA vaccines for COVID-19: what, why and how. Int J Biol Sci. 2021;17(6):1446–60. 33907508 10.7150/ijbs.59233 PMC8071766

[11] Frenck RW, Klein NP, Kitchin N, Gurtman A, Absalon J, Lockhart S, et al. Safety, immunogenicity, and efficacy of the BNT162b2 Covid-19 vaccine in adolescents. N Engl J Med. 2021;385(3):239–50. 34043894 10.1056/NEJMoa2107456 PMC8174030

[12] Lawson-Tovey S, Machado PM, Strangfeld A, Mateus E, Gossec L, Carmona L, et al. SARS-CoV-2 vaccine safety in adolescents with inflammatory rheumatic and musculoskeletal diseases and adults with juvenile idiopathic arthritis: data from the EULAR COVAX physician-reported registry. RMD Open. 2022;8(2):e002322. 35908834 10.1136/rmdopen-2022-002322 PMC9344592

[13] Heshin-Bekenstein M, Ziv A, Toplak N, Hagin D, Kadishevich D, Butbul YA, et al. Safety and immunogenicity of BNT162b2 mRNA COVID-19 vaccine in adolescents with rheumatic diseases treated with immunomodulatory medications. Rheumatology. 2022;61(11):4263–72. 35179569 10.1093/rheumatology/keac103 PMC9383463

[14] Yeo JG, Chia WN, Teh KL, Book YX, Hoh SF, Gao X, et al. Robust neutralizing antibody response to SARS-CoV-2 mRNA vaccination in adolescents and young adults with childhood-onset rheumatic diseases. Rheumatology. 2022;61(11):4472–81. 35199166 10.1093/rheumatology/keac105 PMC8903460

[15] Arslanoglu Aydin E, Baglan E, Bagrul I, Tuncez S, Ozdel S, Bulbul M. Safety of COVID-19 vaccines and disease flares after vaccines in children with rheumatic disease. Postgrad Med. 2022;134(6):616–21. 35535525 10.1080/00325481.2022.2074700

[16] Dimopoulou D, Spyridis N, Vartzelis G, Tsolia MN, Maritsi DN. Safety and tolerability of the COVID-19 messenger RNA vaccine in adolescents with juvenile idiopathic arthritis treated with tumor necrosis factor inhibitors. Arthritis Rheumatol. 2022;74(2):365–6. 34492161 10.1002/art.41977 PMC8653078

[17] Ziv A, Heshin-Bekenstein M, Haviv R, Kivity S, Netzer D, Yaron S, et al. Effectiveness of the BNT162b2 mRNA COVID-19 vaccine among adolescents with juvenile-onset inflammatory rheumatic diseases. Rheumatology. 2023;62(SI2):SI145–51. 35920789 10.1093/rheumatology/keac408 PMC9384675

[18] Akgün Ö, Çakmak F, Guliyeva V, Demirkan FG, Tanatar A, Hançerli Torun S, et al. Humoral response and safety of BNT162b2 mRNA vaccine in children with rheumatic diseases. Rheumatology. 2022;61(11):4482–90. 35353139 10.1093/rheumatology/keac140 PMC9383626

[19] Dimopoulou D, Tsolia MN, Spyridis N, Maritsi DN. Immunogenicity 6 months post COVID-19 mRNA vaccination among adolescents with juvenile idiopathic arthritis on treatment with TNF inhibitors. Rheumatology. 2023;62(SI2):SI205–9. 35788275 10.1093/rheumatology/keac352 PMC9278208

[20] Cerutti A, Rescigno M. The biology of intestinal immunoglobulin A responses. Immunity. 2008;28(6):740–50. 18549797 10.1016/j.immuni.2008.05.001 PMC3057455

[21] Woof JM, Kerr MA. The function of immunoglobulin A in immunity. J Pathol. 2006;208(2):270–82. 16362985 10.1002/path.1877

[22] Wang Z, Lorenzi JCC, Muecksch F, Finkin S, Viant C, Gaebler C, et al. Enhanced SARS-CoV-2 neutralization by dimeric IgA. Sci Transl Med. 2021;13:577. 10.1126/scitranslmed.abf1555 PMC7857415 33288661

[23] Sterlin D, Mathian A, Miyara M, Mohr A, Anna F, Claër L, et al. IgA dominates the early neutralizing antibody response to SARS-CoV-2. Sci Transl Med. 2021;13:577. 10.1126/scitranslmed.abd2223 PMC7857408 33288662

[24] Cox RJ, Brokstad KA, Ogra P. Influenza virus: immunity and vaccination strategies. Comparison of the immune response to inactivated and live, attenuated influenza vaccines. Scand J Immunol. 2004;59(1):1–15. 14723616 10.1111/j.0300-9475.2004.01382.x

[25] Muramatsu M, Yoshida R, Yokoyama A, Miyamoto H, Kajihara M, Maruyama J, et al. Comparison of antiviral activity between IgA and IgG specific to influenza virus hemagglutinin: increased potential of IgA for heterosubtypic immunity. PLoS ONE. 2014;9(1):e85582. 24465606 10.1371/journal.pone.0085582 PMC3895000

[26] Abril AG, Alejandre J, Mariscal A, Alserawan L, Rabella N, Roman E, et al. Titers of IgG and IgA against SARS-CoV-2 proteins and their association with symptoms in mild COVID-19 infection. Sci Rep. 2024;14(1):12725. 38830902 10.1038/s41598-024-59634-y PMC11148197

[27] Ma H, Zeng W, He H, Zhao D, Jiang D, Zhou P, et al. Serum IgA, IgM, and IgG responses in COVID-19. Cell Mol Immunol. 2020;17(7):773–5. 32467617 10.1038/s41423-020-0474-z PMC7331804

[28] Padoan A, Sciacovelli L, Basso D, Negrini D, Zuin S, Cosma C, et al. IgA-Ab response to spike glycoprotein of SARS-CoV-2 in patients with COVID-19: a longitudinal study. Clin Chim Acta. 2020;507:164–6. 32343948 10.1016/j.cca.2020.04.026 PMC7194886

[29] Petty RE, Southwood TR, Manners P, Baum J, Glass DN, Goldenberg J, et al. International league of associations for rheumatology classification of juvenile idiopathic arthritis: second revision, Edmonton, 2001. J Rheumatol. 2004;31(2):390–2. 14760812

[30] Petri M, Orbai A, Alarcón GS, Gordon C, Merrill JT, Fortin PR, et al. Derivation and validation of the systemic lupus international collaborating clinics classification criteria for systemic lupus erythematosus. Arthritis Rheum. 2012;64(8):2677–86. 22553077 10.1002/art.34473 PMC3409311

[31] Jennette JC, Falk RJ, Andrassy K, Bacon PA, Churg J, Gross WL, et al. Nomenclature of systemic vasculitides. Arthritis Rheum. 1994;37(2):187–92. 8129773 10.1002/art.1780370206

[32] Lundberg IE, Tjärnlund A, Bottai M, Werth VP, Pilkington C, de Visser M, et al. 2017 European League Against Rheumatism/American College of Rheumatology classification criteria for adult and juvenile idiopathic inflammatory myopathies and their major subgroups. Ann Rheum Dis. 2017;76(12):1955–64. 29079590 10.1136/annrheumdis-2017-211468 PMC5736307

[33] Roderick MR, Shah R, Rogers V, Finn A, Ramanan AV. Chronic recurrent multifocal osteomyelitis (CRMO) – advancing the diagnosis. Pediatr Rheumatol. 2016;14(1):47. 10.1186/s12969-016-0109-1 PMC5006369 27576444

[34] Gewitz MH, Baltimore RS, Tani LY, Sable CA, Shulman ST, Carapetis J, et al. Revision of the Jones criteria for the diagnosis of acute rheumatic fever in the era of Doppler echocardiography. Circulation. 2015;131(20):1806–18. 25908771 10.1161/CIR.0000000000000205

[35] Kuemmerle-Deschner JB, Ozen S, Tyrrell PN, Kone-Paut I, Goldbach-Mansky R, Lachmann H, et al. Diagnostic criteria for cryopyrin-associated periodic syndrome (CAPS). Ann Rheum Dis. 2017;76(6):942–7. 27707729 10.1136/annrheumdis-2016-209686

[36] Resman Rus K, Korva M, Knap N, Avšič Županc T, Poljak M. Performance of the rapid high-throughput automated electrochemiluminescence immunoassay targeting total antibodies to the SARS-CoV-2 spike protein receptor binding domain in comparison to the neutralization assay. J Clin Virol. 2021;139:104820. 33865031 10.1016/j.jcv.2021.104820 PMC8035809

[37] Poljak M, Oštrbenk Valenčak A, Štrumbelj E, Maver Vodičar P, Vehovar V, Resman Rus K, et al. Seroprevalence of severe acute respiratory syndrome coronavirus 2 in Slovenia: results of two rounds of a nationwide population study on a probability-based sample, challenges and lessons learned. Clin Microbiol Infect. 2021;27(7):e10391–7. 10.1016/j.cmi.2021.03.009 PMC8064903 33838303

[38] Poljak M, Oštrbenk Valenčak A, Štamol T, Seme K. Head-to-head comparison of two rapid high-throughput automated electrochemiluminescence immunoassays targeting total antibodies to the SARS-CoV-2 nucleoprotein and spike protein receptor binding domain. J Clin Virol. 2021;137:104784. 33711693 10.1016/j.jcv.2021.104784 PMC7934695

[39] Udaondo C, Cámara C, Miguel Berenguel L, Alcobendas Rueda R, Muñoz Gómez C, Millán Longo C, et al. Humoral and cellular immune response to mRNA SARS-CoV-2 BNT162b2 vaccine in adolescents with rheumatic diseases. Pediatr Rheumatol. 2022;20(1):64. 10.1186/s12969-022-00724-4 PMC9375068 35964130

